# Ectopic Cushing Syndrome due to Metastatic Medullary Thyroid Cancer

**DOI:** 10.1016/j.aed.2025.03.003

**Published:** 2025-04-10

**Authors:** Majd Oweidat, Anas Abu Rumilah

**Affiliations:** 1 College of Medicine, Hebron University, Hebron, West Bank, Palestine; 2 Division of Endocrinology, Department of Internal Medicine, Al-Ahli Hospital, Hebron, West Bank, Palestine

**Keywords:** ectopic ACTH production, medullary thyroid carcinoma, Cushing syndrome, hypokalemia, thyroid cancer, hypercortisolism

## Abstract

**Background/Objective:**

Cushing syndrome (CS) is a life-threatening condition that occurs due to hypercortisolism. The most common cause of endogenous CS is adrenocorticotropic hormone (ACTH)–dependent CS caused by a pituitary adenoma (Cushing disease). Ectopic production of ACTH from tumors elsewhere in the body is rare and is most often associated with small cell lung cancer. This report describes a patient with ACTH-dependent CS secondary to metastatic medullary thyroid cancer (MTC), a rare cause of ectopic CS.

**Case Report:**

A 52-year-old man presented with progressive diarrhea, fatigue, muscle weakness, and hypokalemia. Initial investigations revealed metabolic alkalosis, elevated urinary potassium excretion, and fasting hyperglycemia. Evaluations confirmed ACTH-dependent hypercortisolism with elevated serum ACTH and urinary free cortisol levels. Imaging and fine needle aspiration confirmed MTC with metastatic lymph node involvement, supported by elevated serum calcitonin and carcinoembryonic antigen levels. Surgical intervention revealed extensive tumor invasion but was complicated by vascular injuries, resulting in postoperative hypoxic brain injury and fatality.

**Discussion:**

This case highlights the importance of early recognition and investigation of unexplained ACTH-dependent hypercortisolism. Delayed diagnosis complicates management, increases morbidity, and worsens outcomes. A systematic diagnostic approach—including biochemical markers, imaging, and cytology—is crucial in identifying rare causes of hypercortisolism.

**Conclusion:**

Early evaluation of persistent hypokalemia and hypercortisolism is essential to prevent delayed diagnosis of CS. This case highlights MTC as a rare source of ectopic ACTH secretion.

**Highlights**

• This is a rare case of Cushing syndrome (CS) caused by ectopic adrenocorticotropic hormone secretion due to metastatic medullary thyroid cancer
• Diagnosis of ectopic CS was supported by nonsuppression of cortisol on high-dose dexamethasone suppression testing and unremarkable pituitary magnetic resonance imaging
• The patient underwent aggressive surgical resection; however, complications from advanced disease led to poor outcomes

**Clinical Relevance**

This case highlights the rare occurrence of ectopic CS due to metastatic medullary thyroid cancer. It highlights the need for early recognition and systematic evaluation of hypercortisolism to improve patient outcomes in complex malignancies.

## Introduction

Cushing syndrome (CS) is a condition characterized by prolonged exposure to high levels of cortisol. This potentially life-threatening condition is linked to serious comorbidities such as hypertension and diabetes. Early diagnosis is crucial to prevent further complications and reduce long-term mortality risks. Endogenous CS can be classified into adrenocorticotropic hormone (ACTH)–dependent (approximately 90%) and ACTH-independent (5%-10%) causes. ACTH-dependent CS is most commonly caused by a pituitary adenoma (approximately 75%), also known as Cushing disease (CD), but can also result from ectopic ACTH production (approximately 15%). Although various tumors can produce ectopic ACTH, small cell lung cancer accounts for the majority of cases. In contrast, ACTH-independent CS is most often caused by adrenal adenomas.^1^, ^2^, ^3^

Diagnosing ectopic CS (ECS) requires a combination of biochemical testing and imaging. In patients with ACTH-dependent CS, an unremarkable pituitary magnetic resonance imaging (MRI) raises suspicion for ECS. High-dose dexamethasone suppression testing (HDDST) and corticotropin-releasing hormone/desmopressin stimulation tests used provide additional clues; however, inferior petrosal sinus sampling (IPSS) remains the “gold standard” to differentiate ECS from CD. Once ECS is suspected, imaging is performed to locate the ectopic ACTH-producing tumor.^4^^,^^5^

Medullary thyroid cancer (MTC) is a rare neuroendocrine tumor that originates from the parafollicular C cells of the thyroid, which are derived from neural crest cells. MTC includes both sporadic and familial forms and accounts for less than 5% of all thyroid cancers but contributes disproportionately to thyroid cancer–related deaths.^6^ In recent literature, MTC has been described in a very few cases as a rare source of ectopic ACTH secretion, with less than 1% of MTC cases exhibiting this feature.^7^ Reported cases highlight the diagnostic challenges and aggressive nature of MTC-induced ectopic ACTH secretion, often presenting with severe hypercortisolism and advanced disease at diagnosis, although this is highly unusual.^8^, ^9^, ^10^, ^11^ The connection between these 2 conditions highlights the importance of considering MTC as a potential cause of ectopic ACTH production in patients with CS. This report describes an unusual case of CS caused by ectopic ACTH secretion due to metastatic MTC.

## Case Report

A 52-year-old man presented with a 1-week history of diarrhea, followed by a month of progressively worsening symptoms, including bilateral lower limb swelling, generalized fatigue, polyuria, and muscle weakness. Initially, he sought medical care and was admitted to a local hospital where routine investigations were performed. The only notable finding was hypokalemia. He was diagnosed with gastroenteritis, treated with intravenous fluids and potassium supplementation, and subsequently discharged. Despite this intervention, his symptoms persisted, with progressive muscle weakness becoming increasingly apparent. Concerned about his condition, he sought further evaluation. At that time, detailed investigations revealed persistent hypokalemia to 2.6 mmol/L, a newly diagnosed type 2 diabetes mellitus (hemoglobin A1C level, 8.5%), and proteinuria. His laboratory findings also showed metabolic alkalosis (bicarbonate level, 32 mmol/L; blood pH level, 7.5), a markedly elevated urinary potassium excretion (50 mmol/L), and fasting cortisol levels within the upper normal range (15 μg/dL). A summary of his key laboratory findings is provided in the Table.

**Table:** Summary of the Patient’s Laboratory Findings

Laboratory test	Value	Normal range
Hemoglobin	12.5 g/dL	13.5-17.5 g/dL (male)
HbA1C	8.50%	<5.7%
Serum ACTH	116 pg/mL	10-60 pg/mL
Potassium	2.6 mmol/L	3.5-5.0 mmol/L
Sodium	140 mmol/L	135-147 mmol/L
Magnesium	0.74 mmol/L	0.65-1.05 mmol/L
Calcium	8.7 mg/dL	9-10.5 mg/dL
Creatinine	0.4 mg/dL	0.6-1.2 mg/dL (male)
Uric acid	1.6 mg/dL	4.0-8.5 mg/dL (male)
Creatine phosphokinase	110 units/L	55-170 units/L
Thyroid-stimulating hormone	1.6 mIU/L	0.4-4 mIU/L
Urine protein	1	Negative
Urine glucose	4	Negative
Urine pH	7	4.5-8.0
Urine potassium	50 mmol/L	40-100 mmol/d
Bicarbonate	32 mmol/L	22-29 mmol/L
Blood pH	7.5	7.35-7.45
Aldosterone/renin ratio	12	<25
Fasting cortisol	15 μg/dL	5-23 μg/dL
Low-dose dexamethasone suppression test	8 mcg/dL	<1.8 μg/dL
High-dose dexamethasone suppression test	12 μg/dL	<1.8 μg/dL
24-h urine free cortisol	400 μg/24 h	<100 μg/24 h
Parathyroid hormone	26.4 pg/mL	10-65 pg/mL
Calcitonin	6114 pg/mL	<30 pg/mL
Carcinoembryonic antigen	245 ng/mL	<5 ng/mL

Abbreviations: ACTH = adrenocorticotropic hormone; HbA1C = hemoglobin A1C.

The underlying cause of his hypokalemia and systemic symptoms remained undiagnosed. He was prescribed eplerenone and potassium supplements, along with oral hypoglycemic agents for newly diagnosed diabetes mellitus, before being discharged. On returning home, his symptoms showed mild improvement; however, hypokalemia recurred with potassium levels dropping to 2.8 mmol/L. He presented to our outpatient clinic for further evaluation, where a detailed history, physical examination, and review of prior investigations were conducted. The patient reported persistent diarrhea, generalized fatigue, progressive muscle weakness, and increasing abdominal girth. He also noted recent facial puffiness and fullness, which were corroborated by comparing his identification photograph with his current appearance. His medical history revealed no prior episodes of hypokalemia, diabetes, or chronic illness. There was no significant family history of endocrine or autoimmune disorders, and he was a nonsmoker with no occupational exposures of relevance. On physical examination, his blood pressure was 120/80 mm Hg, and his body weight was 64 kg, with a body mass index of 23 kg/m^2^. Notable findings included bilateral pitting edema of the lower limbs, facial plethora, puffiness, and thinning of the skin. He showed proximal muscle weakness but had no pathologic bruises or striae. His abdomen appeared distended, although there were no palpable masses. There was no evidence of supraclavicular fullness or a dorsocervical fat pad.

Given his clinical features and unresolved hypokalemia, CS was suspected as the underlying cause of his symptoms. Biochemical confirmation included a fasting cortisol level of 16 μg/dL and a positive overnight dexamethasone suppression test (serum cortisol level, 8 μg/dL; diagnostic threshold, >1.8 μg/dL). The urinary free cortisol levels were significantly elevated at 400 μg/24 hours. The serum ACTH levels were elevated at 116 pg/mL, confirming ACTH-dependent hypercortisolism. Subsequent HDDST revealed nonsuppression of cortisol (12 μg/dL), failing to suppress by more than 50% of the baseline after being given 8 mg of dexamethasone, raising suspicion for an ECS. IPSS and hormone testing such as corticotropin-releasing hormone/desmopressin stimulation tests were not available. Consequently, a combination of biochemical tests and imaging was used for diagnostic evaluation. Pituitary MRI findings were unremarkable, making a pituitary adenoma less likely.

**Fig. 1:**
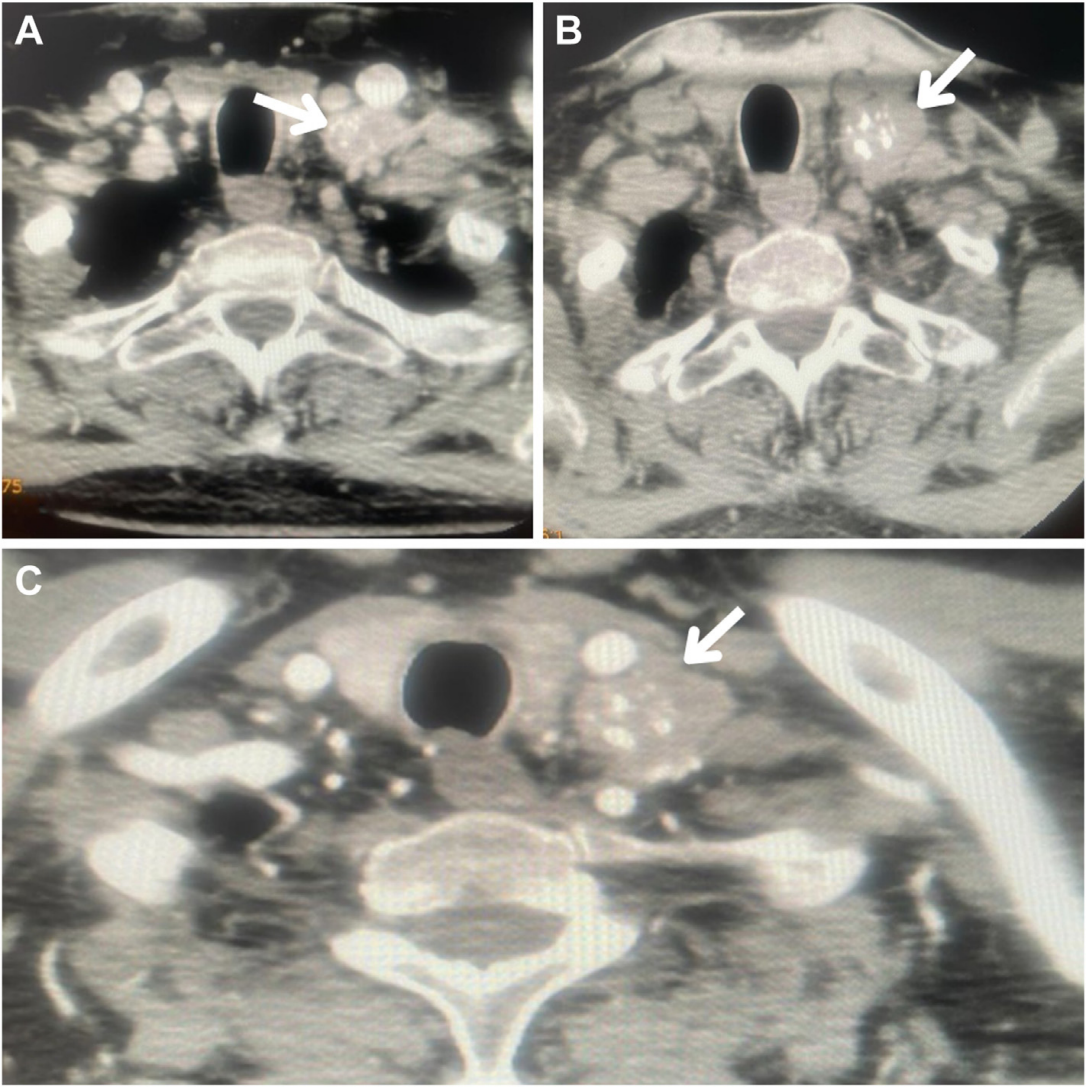
Neck computed tomography showing multiple enlarged lymph nodes seen in the central and left lateral compartments. No pathologic lymph nodes were identified on the right side. White arrows indicate the suspicious lymph nodes in (*A*-*C*).

**Fig. 2:**
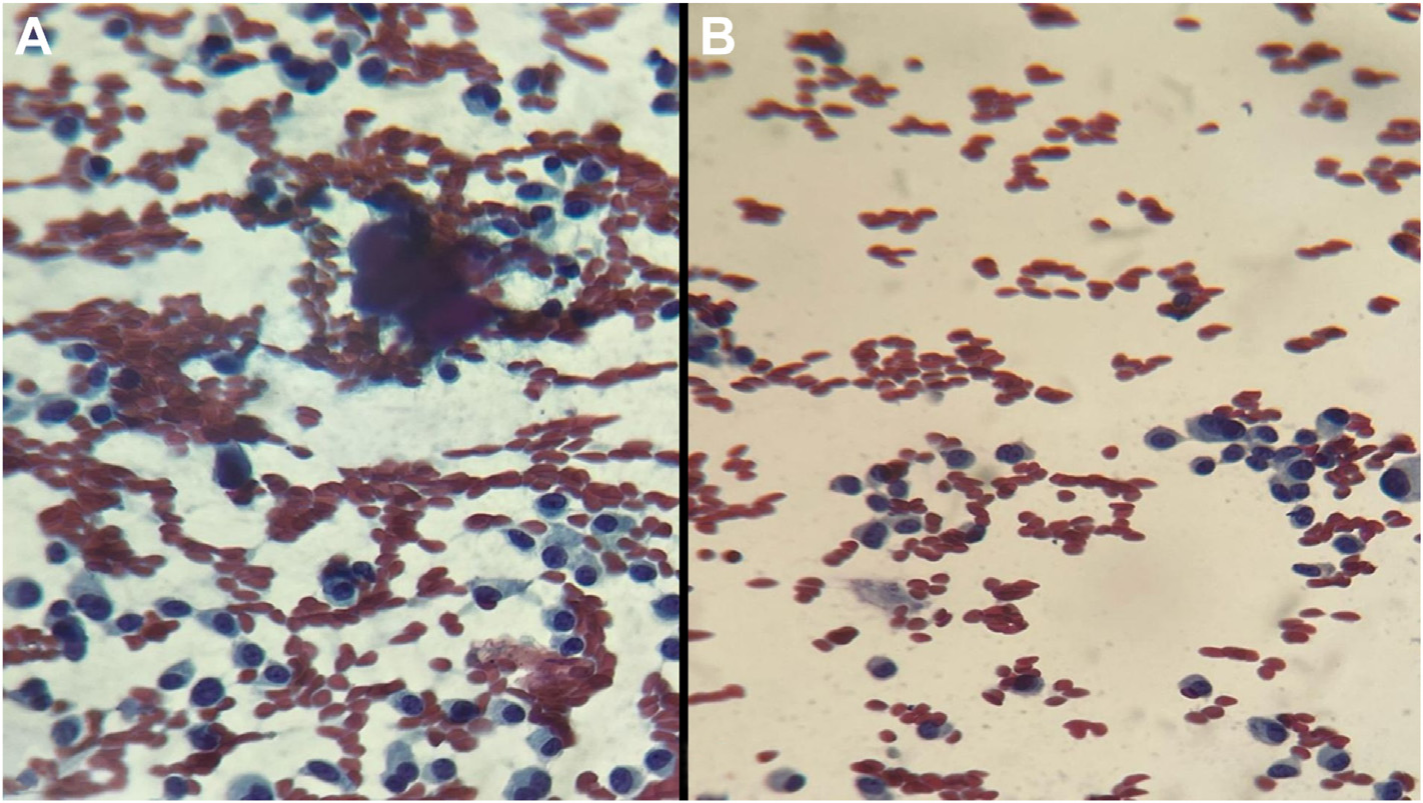
Fine needle aspiration cytology (Papanicolaou stain; magnification, ×10) revealing a cellular smear composed of round, ovoid, and plasmacytoid cells with abundant cytoplasm and eccentric nuclei (*A* and *B*). Notably, amyloid material was present, consistent with the diagnosis of medullary thyroid cancer.

Imaging studies, including neck ultrasound and contrast-enhanced computed tomography (CT) scans of the neck, chest, abdomen, and pelvis, were performed to identify the ectopic source. Neck CT revealed multiple rounded enlarged lymph nodes, measuring up to 1.4 cm in the central compartment and up to 2.3 × 2.6 cm in the left lateral compartment, with no pathologic lymph nodes on the right side, as shown in Figure 1. Neck ultrasound revealed a hypoechoic thyroid nodule in the left lobe (1 × 0.9 × 1.1 cm) with suspicious features, including microcalcifications and peripheral vascularity, alongside enlarged lymph nodes. Fine needle aspiration cytology was performed on the thyroid nodule, central compartment lymph nodes, and left lateral lymph nodes, confirming MTC with metastatic involvement of the lymph nodes, as illustrated in Figure 2. Further biochemical evaluation supported the diagnosis of MTC, with a calcitonin level of 6114 pg/mL and carcinoembryonic antigen level of 245 ng/mL. Screening for multiple endocrine neoplasia type 2 revealed normal parathyroid hormone levels, calcium levels, and 24-hour urine metanephrines and catecholamines.

Whole-body positron emission tomography (PET)/CT using 18-fluorodeoxyglucose (18F-FDG) was performed to assess for distant metastases. The 18F-FDG PET/CT showed no findings beyond regional lymph node involvement. Although 18F-FDG PET/CT is not the optimal imaging modality for detecting MTC metastases in such case, it was the most readily available option in our center and region. More reliable modalities such as gallium-68-DOTATATE PET/CT were considered but were not accessible due to resource limitations. Given the concern for MTC progression, the patient opted to proceed directly with surgical management, including total thyroidectomy and extensive neck lymph node dissection, rather than initiating medical therapy for hypercortisolism preoperatively.

The patient underwent total thyroidectomy with bilateral central compartment (levels VI and VII) and bilateral functional lateral compartment (levels II-IV) neck dissection at another hospital. Intraoperatively, the tumor showed an aggressive growth pattern, invading critical structures, including the recurrent laryngeal nerves, apical pleura, left internal jugular vein, vertebral plane, and subclavian vessels. Due to this extensive invasion, the surgical resection was challenging and incomplete. The surgery was complicated by vascular and pleural injuries, including injury to the subclavian vessels, which required urgent repair by the vascular surgery team, and left pleural injury, which was repaired intraoperatively. Postoperatively, the patient developed significant bleeding and hematoma formation, necessitating wound reopening for hemostatic control. Tragically, the patient experienced a hypoxic brain injury and passed away on the same day as the reoperation. Due to these intraoperative complications and the incomplete tumor resection, postoperative ACTH and cortisol levels were not reassessed, and further follow-up imaging was not conducted.

## Discussion

The most common cause of hypercortisolism is exogenous (iatrogenic) CS, typically due to prolonged glucocorticoid therapy. This leads to hypercortisolism, suppression of ACTH, and subsequent bilateral adrenal atrophy. Endogenous CS, on the other hand, is most commonly ACTH-dependent, primarily caused by pituitary adenomas (CD). ACTH-dependent CS can also result from ectopic ACTH production, often associated with malignancies such as small cell lung cancer, renal cell carcinoma, pancreatic or bronchial carcinoid tumors, pheochromocytoma, or MTC.^1^^,^^2^ This patient’s presentation highlights the uniqueness of this case, where hypercortisolism led to the discovery of metastatic MTC. The mechanism behind ACTH production in MTC is not well understood; however, the most acceptable proposed mechanism is the direct secretion of ACTH by the tumor cells.^12^ These tumors tend to be aggressive, and metastasis to distant sites can complicate the condition.

Diagnosing hypercortisolism requires the presence of at least 2 abnormal results among the 24-hour urine free cortisol, low-dose dexamethasone suppression test, and late-night salivary cortisol, or markedly elevated 24-hour urine free cortisol level on at least 2 occasions. The plasma ACTH levels help distinguish between ACTH-dependent CS (>20 pg/mL) and ACTH-independent CS (<5 pg/mL).^1^^,^^5^ HDDST results raised strong suspicion for the ECS. IPSS is the “gold standard” to differentiate ECS from CD according to the Pituitary Society guidelines.^5^ However, in this case, HDDST was used due to resource limitations and the unavailability of IPSS. Additionally, given the ongoing geopolitical situation and associated travel restrictions, the patient was unable to seek specialized testing abroad. Pituitary MRI, as the first-line imaging modality for CD, was performed and found unremarkable, making a pituitary adenoma less likely but not entirely ruling it out.^5^ This result helped narrow the differential diagnosis, as the failure of cortisol suppression on HDDST, combined with the unremarkable MRI, strongly suggested ECS. The severity and rapid onset of this patient’s symptoms, including profound hypokalemia, metabolic alkalosis, and progressive muscle weakness, combined with the unremarkable MRI and nonsuppressible cortisol on HDDST, strongly supported the diagnosis of ECS. Further imaging, including neck ultrasound and whole-body contrast-enhanced CT scans, provided critical insights. These findings, combined with fine needle aspiration cytology confirming MTC with metastatic lymph node involvement, facilitated the diagnosis. Such a diagnosis emphasizes the importance of a multidisciplinary stepwise approach involving surgery, oncology, and endocrinology. This approach enabled endocrinologists to diagnose hypercortisolism, oncologists to confirm metastatic MTC, and surgeons to attempt tumor resection.

The management of CS depends on the underlying cause, with curative surgery being the first-line treatment for endogenous CS. For adrenal tumors, adrenalectomy is performed, whereas CD is treated with transsphenoidal hypophysectomy.^1^ In cases of ectopic ACTH production, the primary goal is to identify and resect the culprit tumor, often requiring extensive surgery with lymph node dissection, as appropriate.^1^ However, tumor resection can be challenging, especially in advanced metastatic disease, and hypercortisolism itself in such cases should often be managed first with medical therapy or bilateral adrenalectomy to prevent life-threatening complications.^1^ Standard MTC treatment includes total thyroidectomy and neck dissection, with additional therapies such as external beam radiation or systemic treatments for metastatic disease.^13^ Surgical resection aimed to remove the primary tumor and metastases; however, the aggressive nature of MTC led to complications, which are common in advanced cases due to tumor invasion into critical structures.^13^ When surgical cure is not possible, chemotherapy or targeted therapies may be used to control tumor progression and hormone secretion.^14^ Unlike well-differentiated thyroid cancers, MTC does not respond to radioactive iodine ablation or thyroid-stimulating hormone suppression therapy.^14^

Hypercortisolism affects multiple organ systems, increasing the risk of hypertension, cardiovascular disease, diabetes, impaired wound healing, and coagulopathy, all of which may contribute to poor surgical outcomes. Additionally, muscle weakness, infections, and impaired immune response further complicate postoperative recovery.^15^ Given these effects, it is likely that the patient’s hypercortisolism in this article contributed to the perioperative complications and ultimately the poor surgical outcome.

Few published cases highlight the exceptional rarity and diagnostic challenges of MTC-induced CS.^7^^,^^16^ Reported cases share common themes: patients often presented with hypercortisolism, hypokalemia, and a thyroid mass. Despite surgical and medical interventions, outcomes were poor in most cases due to aggressive metastatic MTC and limited treatment efficacy. A review of 1640 MTC cases found ectopic ACTH secretion in only 0.6% to 0.7% of patients, with most presenting at an advanced stage and 90% having distant metastases at diagnosis. In several cases, clinical features of CS, such as hypokalemia, hypertension, and cushingoid appearance, were common but nonspecific, delaying recognition of the underlying malignancy.^7^ A case involved a 67-year-old man with hypercortisolism due to ectopic ACTH secretion from metastatic MTC, presenting with myopathy, hypokalemia, and elevated calcitonin level. Despite treatment, the patient succumbed to complications.^8^ Another case of a 41-year-old man with ectopic ACTH secretion and hypercortisolism was found to have metastatic tumor originating from MTC. Despite a normal thyroid ultrasound result, elevated serum calcitonin levels confirmed the MTC diagnosis.^10^ Another case was a 54-year-old man with ectopic ACTH secretion secondary to MTC, which was confirmed by immunohistochemistry. Treatment included ketoconazole, chemotherapy, and targeted therapy with sunitinib; however, disease progression led to hepatic metastases and ultimately death.^11^ These cases highlight the diagnostic complexity of ECS in MTC, reflecting the aggressive nature and treatment challenges of metastatic MTC.

This case highlights the importance of recognizing CS as a potential cause of unexplained hypokalemia, which can serve as an early clue to hypercortisolism.^1^ A delay in diagnosing CS can lead to prolonged morbidity and missed opportunities for early intervention.^1^ This case highlights ethical concerns in surgical risk assessment and complications. Multidisciplinary evaluation balanced benefits and risks, emphasizing clear patient-family communication. This article has limitations, including the inability to generalize findings and the absence of long-term follow-up data.

## Conclusion

This case presents a very rare and complex presentation of CS due to ectopic ACTH secretion from metastatic MTC. Clinicians should be aware that ectopic ACTH production can originate from various malignancies, including MTC, even in the absence of overt thyroid disease. Early recognition and intervention are critical in improving prognosis in such aggressive malignancies.

## Disclosure

The authors have no conflicts of interest to disclose.
